# Heart failure and suicide mortality and suicide attempts: a systematic review and meta-analysis of cohort studies

**DOI:** 10.3389/fcvm.2026.1888860

**Published:** 2026-08-28

**Authors:** Ting Meng, Rui Zhang, Qiufeng Li, Yaqin Li, Hui Zhou, Jian Zhang

**Affiliations:** 1 Department of Pharmacy, West China Hospital, Sichuan University, Chengdu, China; 2 Chengdu Women's and Children's Central Hospital, School of Medicine, University of Electronic Science and Technology of China, Chengdu, China; 3 Sichuan Provincial Rehabilitation Hospital, Chengdu, China

**Keywords:** cohort studies, heart failure, meta-analysis, risk, suicide-related outcomes

## Abstract

**Objective:**

This systematic review and meta-analysis aimed to synthesize longitudinal observational evidence on the associations of heart failure (HF) with suicide mortality and suicide attempts.

**Methods:**

PubMed, Embase, and the Cochrane Library were systematically searched from inception to April 11, 2026, to identify cohort studies evaluating suicide mortality or suicide attempts among individuals with HF. Study quality was assessed using the Newcastle-Ottawa Scale. Because suicide mortality and suicide attempts are clinically distinct outcomes, they were analyzed separately. The primary quantitative synthesis was restricted to studies evaluating suicide death or completed suicide and reporting adjusted hazard ratios (HRs). Adjusted HRs were log-transformed and pooled using a generic inverse-variance random-effects model. Studies evaluating suicide attempts were summarized narratively because only two studies were available and they differed substantially in population, clinical setting, and effect measure. Between-study heterogeneity was assessed using Cochran's *Q* test and the I² statistic.

**Results:**

Seven cohort studies published between 2018 and 2026, involving 17,696,608 participants, were included in the systematic review. Five studies evaluating suicide death or completed suicide contributed to the primary meta-analysis. HF was associated with suicide mortality, with a pooled adjusted HR of [HR] (95% CI: [lower–upper]; I^2^ = [I^2^]%, *P* for heterogeneity = [*P* value]). Two studies evaluated suicide attempts but were not pooled because one reported an HR in a large community-based cohort, whereas the other reported an RR in a small, highly selected population of patients with end-stage HF undergoing left ventricular assist device implantation. No included study reported suicidal ideation as an independently extractable longitudinal outcome.

**Conclusion:**

Observational cohort evidence suggests that HF is associated with an increased hazard of suicide mortality. Evidence regarding suicide attempts remains too limited and heterogeneous for a reliable pooled estimate, and no independently extractable longitudinal evidence was available for suicidal ideation. Residual confounding, between-study heterogeneity, and the small number of eligible studies limit causal interpretation and the certainty of the findings.

## 1 Introduction

Heart failure (HF) is a complex clinical syndrome characterized by the structural or functional inability of the heart to pump sufficient blood to meet the body's metabolic demands, leading to debilitating symptoms such as dyspnea, fatigue, and severe fluid retention (1). As the terminal stage of various cardiovascular diseases, HF has reached pandemic proportions, currently affecting over 64 million people worldwide. The condition remains a leading cause of frequent hospitalizations and mortality, posing a tremendous socioeconomic and clinical burden on global healthcare systems (2). Despite remarkable advancements in pharmacological treatments—such as the widespread application of foundational therapies (e.g., ARNIs, beta-blockers, and SGLT2 inhibitors)—and device-based interventions, the prognosis for HF remains unacceptably poor. The 5-year survival rate is comparable to, and sometimes worse than, many advanced malignancies (3). Furthermore, HF is highly heterogeneous, encompassing distinct phenotypes (e.g., HFpEF and HFrEF) and commonly co-occurring with systemic complications like renal dysfunction and cognitive impairment, which historically directed clinical focus almost exclusively toward physical stabilization and hemodynamic management (4).

Beyond its physical manifestations, HF has been consistently associated with adverse mental health outcomes. Patients with HF frequently experience depression, anxiety, psychological distress, impaired sleep, and reduced quality of life compared with individuals without HF (5). Several biological and psychosocial pathways have been proposed to explain this association. For example, systemic inflammation, neurohormonal activation, autonomic dysfunction, sleep disturbance, cerebral hypoperfusion, and cognitive impairment may contribute to psychological vulnerability in some patients with HF (6). Psychosocial factors, including symptom burden, loss of functional independence, repeated hospitalizations, financial strain, and perceived burdensomeness, may further exacerbate distress (7). However, these pathways have not been directly tested in most large-scale epidemiological cohorts and should therefore be regarded as plausible explanatory hypotheses rather than established causal mechanisms. Mental health problems in HF are also clinically relevant because they are associated with poorer self-care, reduced medication adherence, and worse clinical outcomes (8).

Suicide and suicide attempts are major public health concerns, influenced by psychiatric disorders, previous self-harm, substance use, socioeconomic adversity, social isolation, access to healthcare, and physical illness (9). Chronic cardiovascular diseases may identify populations with increased vulnerability to suicide-related outcomes, but the extent to which HF itself contributes independently to this risk remains uncertain. Existing cohort studies have reported inconsistent findings: some have observed elevated suicide risk after HF diagnosis, whereas others suggest that the association may be attenuated after adjustment for psychiatric comorbidity, socioeconomic factors, self-harm history, or other somatic diseases (10). In addition, suicide-related outcomes are not uniformly defined across studies. Completed suicide or suicide death, suicide attempts, suicidal ideation, and composite suicide-related outcomes are related but clinically distinct endpoints with different epidemiology, determinants, ascertainment methods, and degrees of underreporting. Therefore, the term “suicide-related outcomes” is used only as an umbrella term in the systematic review. For quantitative synthesis, suicide mortality and suicide attempts were treated as separate outcome categories and were not assumed to represent a single homogeneous clinical endpoint.

To address these knowledge gaps, we conducted a systematic review of cohort studies examining the associations of HF with suicide mortality and suicide attempts. Because these endpoints are clinically distinct, we used an outcome-specific analytical framework. Studies evaluating suicide death or completed suicide and reporting adjusted HRs were included in the primary quantitative synthesis, whereas studies evaluating suicide attempts were summarized separately. We did not conduct subgroup analyses by geographic region or study design because only one study represented the Asian population and only one used a prospective design, precluding meaningful subgroup comparisons. Because all included studies were observational, our objective was to summarize epidemiological associations rather than establish causality or validate specific biological mechanisms.

## 2 Materials and methods

This meta-analysis was conducted in accordance with the guidelines of the Preferred Reporting Items for Systematic Reviews and Meta-Analyses (PRISMA) (11). The protocol was pre-registered in the International Prospective Register of Systematic Reviews (PROSPERO) platform, and the approval number is CRD420261387801.

### 2.1 Data sources and searches

PubMed, Embase, and Cochrane Library were searched for studies published from database inception to April 11, 2026. There were no language restrictions, and the search strategy combined the use of medical subject headings (MeSH) and keywords. The search terms included: heart failure, suicide and risk. The full search strategy is included in Supplementary Material. The reference lists of included cohort studies and other published meta-analyses were also examined to identify relevant trials.

### 2.2 Eligibility criteria

Inclusion criteria

Studies were considered eligible if they met the following criteria based on the PICOS framework:

(a) Participants: Individuals with a clear clinical diagnosis of heart failure who were monitored or assessed for subsequent suicidal behavior. Given the epidemiology of heart failure, this primarily focuses on adult populations;
(b) Exposure: Patients diagnosed with heart failure (HF), encompassing any clinical subtypes (e.g., heart failure with preserved ejection fraction, heart failure with reduced ejection fraction, chronic or acute heart failure);
(c) Comparator: Healthy individuals, matched controls, or the general population without a diagnosis of heart failure;
(d) Outcome: Eligible outcomes included suicide death or completed suicide, suicide attempt or self-harm with suicidal intent, suicidal ideation, and composite suicide-related outcomes, as defined by the original studies. Outcome definitions were extracted exactly as reported. For synthesis, the outcomes were classified into clinically distinct categories. Suicide death and completed suicide were grouped under the fatal-outcome category of suicide mortality, while suicide attempts were treated as a separate nonfatal outcome. These categories were not statistically combined. In the final set of included studies, five studies reported suicide death or completed suicide and two reported suicide attempts. No included study reported suicidal ideation as an independently extractable longitudinal outcome. When multiple estimates were available for the same eligible outcome, we extracted the most fully adjusted estimate.
(e) Study Design: Observational cohort studies (prospective or retrospective) or cohort-based nested case-control studies;
(f) Others: No language or regional restrictions were applied in the selection of studies to ensure a comprehensive and global representation of the data.

Exclusion criteria

Studies were excluded if they met any of the following criteria:

(a) Publication types such as conference abstracts, letters to the editor, case reports, editorials, or review articles where original data could not be extracted;
(b) Studies with incomplete data, relying solely on unadjusted raw estimates, or lacking the specific suicide-related outcomes of interest;
(c) Studies where heart failure was assessed as a composite exposure combined with other cardiovascular diseases (e.g., acute myocardial infarction, stroke, or general “coronary heart disease”), making it impossible to independently extract the specific effect size for heart failure.

### 2.3 Study selection

Study selection was performed by two reviewers (Ting Meng and Rui Zhang) who independently screened the literature based on the eligibility and exclusion criteria. Duplicate and irrelevant articles were first excluded according to their titles and abstracts. Thereafter, the full texts of the potentially eligible articles were downloaded and read to identify all eligible studies. Any disagreements were resolved by the third reviewer (Jian Zhang), who acted as an arbiter.

### 2.4 Data extraction

Data extraction was performed independently by the two above-mentioned reviewers (Ting Meng and Rui Zhang) who consulted the guidelines on data extraction for systematic reviews and meta-analysis. A standardized data extraction form was used to collect the following information: first author, publication year, country or region, study design, data source, participant age, follow-up duration, HF diagnostic criteria, sample size, type and definition of suicide-related outcome, outcome ascertainment method, number of suicide-related events when available, adjusted effect estimate with 95% CI, type of effect measure reported by the original study, and covariates included in the adjustment model. Disagreements were resolved by discussion with Jian Zhang to reach a consensus.

### 2.5 Risk of bias assessment

The Newcastle-Ottawa scale (NOS) was used to assess the quality of included studies (12). Stars ranged from 0 to 9 points for cohort studies, four stars for selection of participants and measurement of exposure, two stars for comparability, and three stars for assessment of outcomes and adequacy of follow-up, with more stars indicating higher quality of study. Scores of 0–3, 4–6, and 7–9 were considered to indicated low, moderate, and high quality, respectively.

### 2.6 Statistical analysis

Suicide mortality and suicide attempts were analyzed separately because they are clinically distinct outcomes. The primary quantitative synthesis was restricted to studies evaluating suicide death or completed suicide and reporting adjusted HRs. Suicide death and completed suicide were treated as fatal outcomes under the category of suicide mortality, while the original definitions and ascertainment methods were retained at the study level.

For the primary meta-analysis, the most fully adjusted HR and corresponding 95% CI from each eligible study were logarithmically transformed and pooled using a generic inverse-variance random-effects model. The pooled result was back-transformed and reported as a pooled HR, rather than as an RR. Between-study heterogeneity was quantified using Cochran's *Q* test and the I^2^ statistic. The random-effects model was used because variation in the underlying association was expected across populations, healthcare systems, follow-up periods, and adjustment strategies.

Studies evaluating suicide attempts were not combined with studies evaluating suicide mortality. The suicide-attempt studies were also not statistically pooled with each other because only two studies were available, they enrolled markedly different populations, and they reported different effect measures. Their adjusted estimates were therefore summarized narratively. A separate synthesis of suicidal ideation was not possible because no included study reported this outcome as an independently extractable longitudinal endpoint.

Leave-one-out sensitivity analysis was performed for the primary suicide-mortality meta-analysis by sequentially omitting each study. The resulting pooled HRs and heterogeneity statistics are provided in the Supplementary Material. Meta-regression was not performed because only five studies contributed to the primary meta-analysis. Subgroup analyses by geographic region and study design were not performed because only one study was conducted in Asia and only one study used a prospective design; consequently, statistically meaningful subgroup comparisons were not possible.

Formal assessment of funnel plot asymmetry, Egger's regression, and trim-and-fill analysis was not performed because only five studies contributed to the primary meta-analysis. With so few studies, these methods have low statistical power and may produce unstable or misleading results, particularly in the presence of between-study heterogeneity. All statistical analyses were performed using Stata version 14.0 (Stata Corp, College Station, TX, USA).

## 3 Results

### 3.1 Literature search

**Figure 1:**
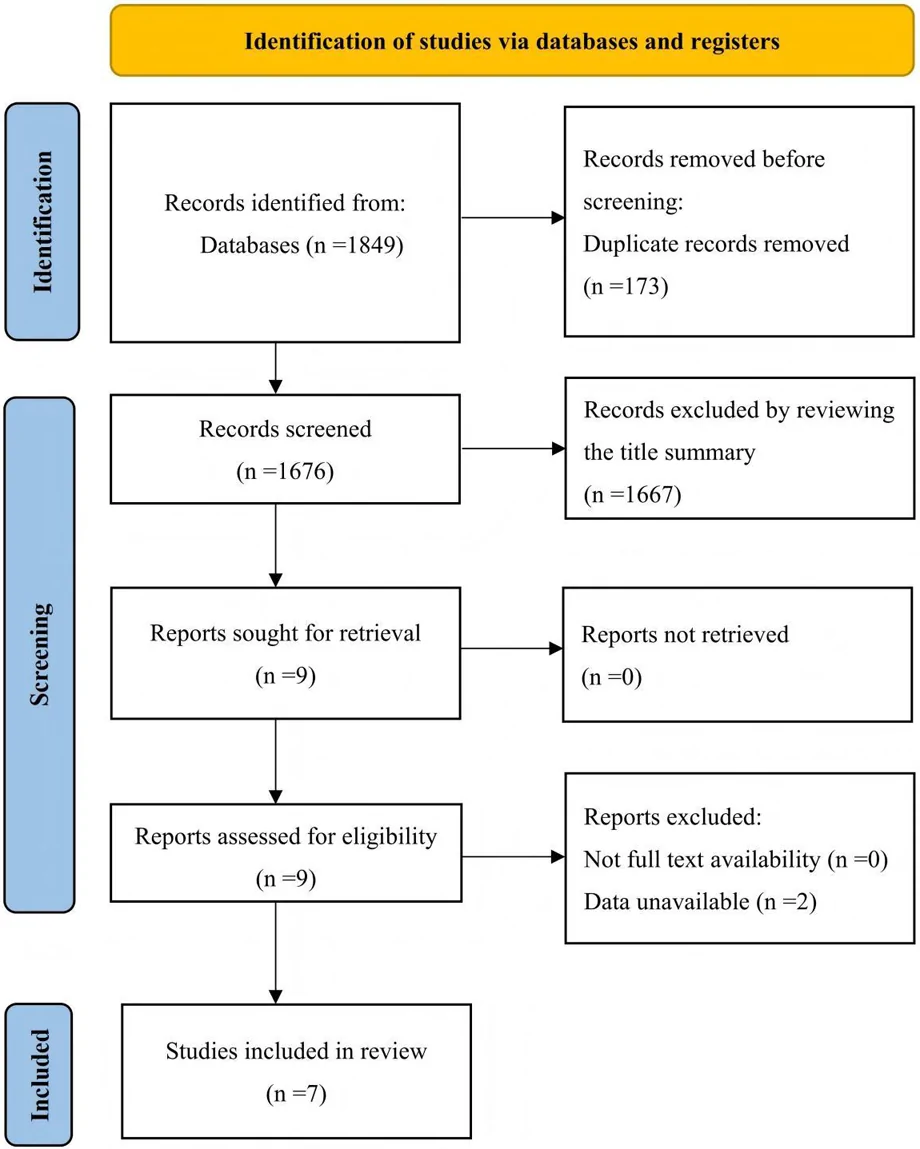
PRISMA flow diagram of study selection.

The database search identified 1,849 records. After removal of 173 duplicates, 1,676 records were screened by title and abstract. Of these, 1,667 records were excluded as irrelevant. Nine full-text articles were assessed for eligibility, and two were excluded because the required data were unavailable. Finally, seven cohort studies were included in the systematic review and meta-analysis (8, 13–18). The study selection process is shown in Figure 1.

### 3.2 Study characteristics

Seven cohort studies published between 2018 and 2026 were included, comprising 17,696,608 participants. Most studies were conducted in European countries, including Sweden, Denmark, the United Kingdom, and France, and one study was conducted in Taiwan. Six studies used retrospective cohort designs based on national registers, administrative databases, or large population-based cohorts, whereas one study used a prospective design and enrolled patients with end-stage HF requiring left ventricular assist device implantation. The geographic and methodological distribution of the evidence was imbalanced, with only one study conducted in Asia and only one prospective cohort.

HF was primarily identified using ICD codes, clinical registers, outpatient records, primary care records, or clinical criteria. Five studies assessed fatal outcomes reported as suicide death or completed suicide, generally using national mortality registries or cause-of-death records. These five studies reported adjusted HRs and were included in the primary meta-analysis of suicide mortality.

Two studies assessed suicide attempts and were analyzed separately. Yang et al. evaluated incident suicide attempts in a large community-based UK Biobank cohort and reported an adjusted HR. Charton et al. evaluated suicide attempts among 494 patients with end-stage HF undergoing LVAD implantation and reported an adjusted RR. Because suicide attempts are clinically distinct from suicide mortality, and because these two studies differed markedly in population, clinical setting, and effect measure, they were not statistically pooled. No included study reported suicidal ideation as an independently extractable longitudinal outcome.

Outcome ascertainment was based on national death registries, administrative health records, hospital records, or prospective clinical follow-up. Follow-up duration ranged from 1.6 to 13.8 years when reported. Although all included studies provided adjusted estimates, the covariates included in the adjustment models varied substantially across cohorts, including demographic characteristics, psychiatric history, socioeconomic factors, comorbidities, and lifestyle factors. The study-level outcome definitions, ascertainment methods, adjusted effect estimates, and analytical classifications are summarized in Table 1.

**Table 1:** Characteristics and outcome-specific analytical classification of the included studies.

Author	Year	Country	Study type	Age (years)	Follow-years	Diagnostic criteria	Outcome definition	Outcome ascertainment	Analytical treatment	Effect measure reported	Sample size	Confounders adjusted
Crump.	2026	Sweden	Retrospective Cohort Study	64 (IQR, 56–72)	12	ICD-8, ICD-9, ICD-10, Outpatient Registers, primary care records	Suicide death/completed suicide	Swedish national registers, including cause-of-death data	Included in the primary suicide-mortality meta-analysis	Adjusted HR	537,802	Age, sex, birth country, marital status, education level, and prior diagnosis of mental disorders
Yang.	2024	UK	Retrospective Cohort Study	63.0 (57.0–69.0)	7.6	ICD-10	Suicide attempt	UK Biobank-linked hospital and health records; incident suicide attempt identified from linked clinical records	Narrative synthesis of suicide attempts	Adjusted HR	191,768	Demographic characteristics (age, sex, and race), socioeconomic characteristics (Townsend deprivation index, educational attainment, and annual household income), and lifestyle factors (smoking status and alcohol drinking status)
Østergaard.	2024	Denmark	Retrospective Cohort Study	All ages	/	ICD-10	Suicide death/completed suicide	Danish national registers/cause-of-death registry	Included in the primary suicide-mortality meta-analysis	Adjusted HR	6,635,857	Sex, age, birth year, and history of mental disorders
Crump.	2022	Sweden	Retrospective Cohort Study	64.9 (18.5–75.0)	13.8	ICD-10	Suicide death/completed suicide	Swedish national registers/cause-of-death data	Included in the primary suicide-mortality meta-analysis	Adjusted HR	1,700,292	Birth year, sex, birth country, marital status, education level, income, myocardial infarction, alcohol use disorder, cancer, chronic obstructive pulmonary disease (COPD), diabetes, and Charlson comorbidity index score
Petersen.	2020	Denmark	Retrospective Cohort Study	≥15	/	ICD-8, ICD-10	Suicide death/completed suicide	Danish nationwide registers/cause-of-death registry	Included in the primary suicide-mortality meta-analysis	Adjusted HR	7,298,002	Sex, period, age group, living status,income level, Charlson Comorbidity Index, psychiatric disorders prior to heart disease and self-harm prior to heart disease
Charton.	2020	France	Prospective Cohort Study	58.9 (50.3–65.8)	1.6	End-stage heart failure (Stage D) requiring Left Ventricular Assist Device (LVAD) implantation.	Suicide attempt	Prospective clinical follow-up within ASSIST-ICD/clinical records	Narrative synthesis of suicide attempts	Adjusted RR	494	Age, sex, ethnic origin, education level, marital status and household income
Wu.	2018	Taiwan	Retrospective Cohort Study	≥15	/	ICD-9-CM	Suicide death/completed suicide	Taiwan national claims and mortality records; death registry linkage	Included in the primary suicide-mortality meta-analysis	Adjusted HR	212,206, 1,120,187	Hypertension, cancer, psychiatric comorbidities duet to close associations with suicide: substance use disorders, anxiety disorders, mood disorders, and psychotic-related disorders

### 3.3 Quality assessment

The methodological integrity of the included cohort studies was evaluated using the Newcastle-Ottawa Scale (NOS). The appraisal yielded a mean score of 7.3 across all literature. Notably, no individual study achieved a score lower than 6, demonstrating that the synthesized evidence possesses moderate methodological quality. The detailed itemized scores for each investigation are summarized in Table 2.

**Table 2:** NOS scores of the included studies.

Study	Year	Selection	Comparability	Outcome	Total
Cohort studies (*n* = 7)					
Crump, et al	2026	****	**	**	8
Yang, et al	2024	****	**	**	8
Østergaard, et al	2024	****	**	**	8
Crump, et al	2022	***	**	**	7
Petersen, et al	2020	***	**	**	7
Charton, et al	2020	***	**	*	6
Wu, et al	2018	***	**	**	7

### 3.4 Association between heart failure and suicide mortality

**Figure 2:**
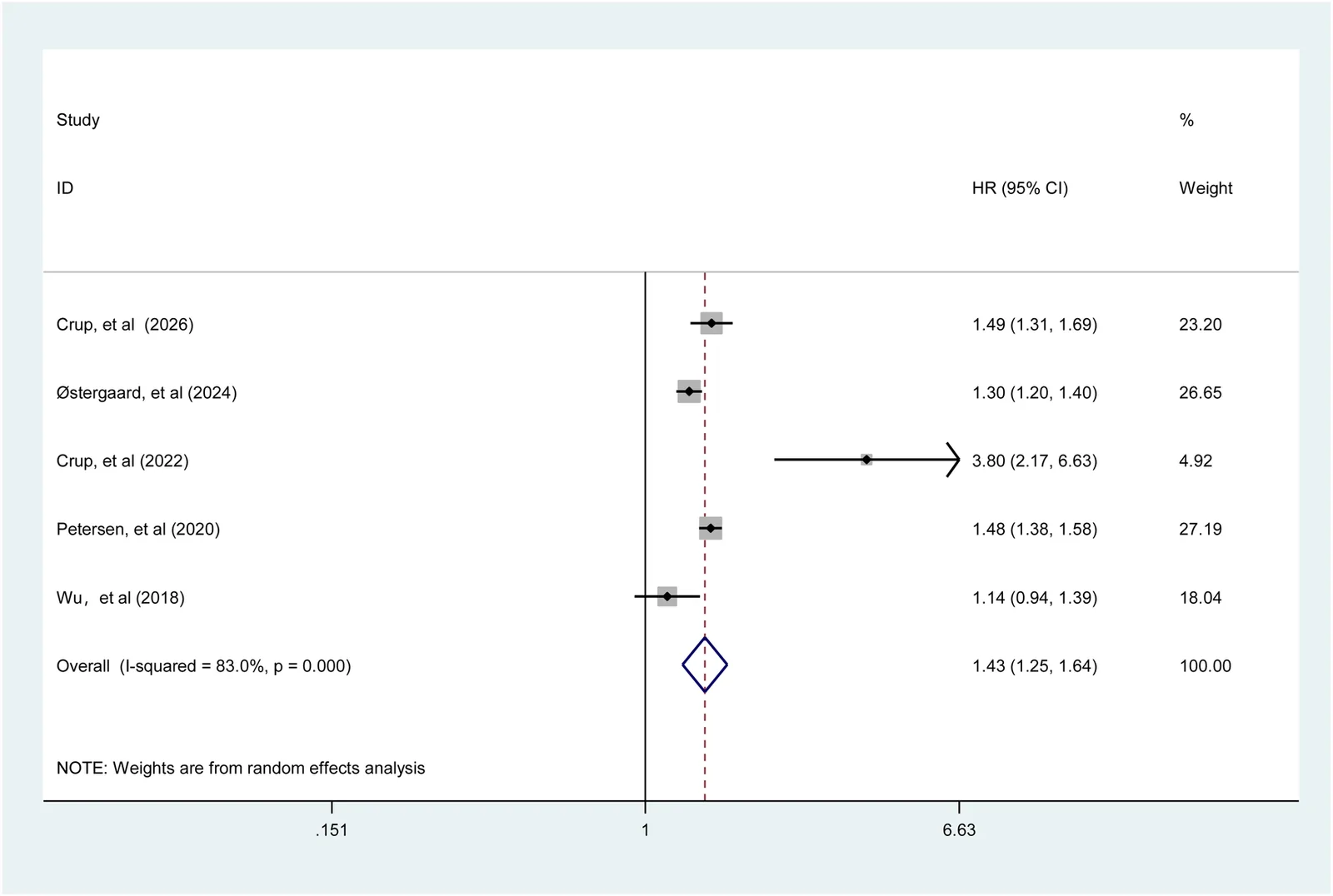
Random-effects meta-analysis of adjusted hazard ratios for suicide mortality among individuals with heart failure.

Five cohort studies evaluating suicide death or completed suicide and reporting adjusted HRs were included in the primary meta-analysis (8, 13, 15, 16, 18). In the random-effects model, HF was associated with a higher hazard of suicide mortality, with a pooled adjusted HR of 1.43 (95% CI: 1.25–1.64; *P* < 0.001; Figure 2). Substantial between-study heterogeneity was observed (I^2^ = 83.0%, *P* for heterogeneity <0.001). The pooled HR should be interpreted as an average time-to-event association across observational cohorts rather than as evidence of a causal effect. Differences in healthcare systems, patient characteristics, HF severity, follow-up duration, outcome ascertainment, and covariate adjustment may have contributed to the observed heterogeneity.

In the leave-one-out sensitivity analysis, the pooled adjusted HRs ranged approximately from 1.37 to 1.51 after sequential omission of individual studies. The lowest pooled HR was observed after omission of Crump et al. (8), whereas the highest was observed after omission of Østergaard et al. (15). All leave-one-out pooled estimates remained above the null value, and their 95% CIs did not include 1.00, indicating that the positive association between HF and suicide mortality was not driven by any single study. Nevertheless, the sensitivity analysis does not explain or eliminate the substantial between-study heterogeneity. The complete leave-one-out results are presented in Supplementary Figure S1.

### 3.5 Association between heart failure and suicide attempts

Two studies evaluated suicide attempts and were not statistically pooled. Yang et al. reported an adjusted HR of 3.00 (95% CI: 1.01–8.90) in a large community-based cohort. Charton et al. reported an adjusted RR of 2.58 (95% CI: 1.47–4.53) among 494 patients with end-stage HF undergoing LVAD implantation. These estimates should not be directly compared because the studies differed substantially in population, HF severity, clinical setting, follow-up, and effect measure. Consequently, the available evidence is insufficient to estimate a common quantitative association between HF and suicide attempts.

### 3.6 Assessment of reporting bias

Only five studies contributed to the primary meta-analysis of suicide mortality. Therefore, funnel plot inspection, Egger's regression, and trim-and-fill analysis were not performed because these methods would have low power and could yield unstable or misleading results with so few studies. Publication bias and selective reporting cannot be excluded, but they could not be reliably quantified in the present analysis.

## 4 Discussion

### 4.1 Main findings

This systematic review included seven cohort studies involving 17,696,608 participants. In the outcome-specific quantitative synthesis of five studies, HF was associated with suicide mortality, with a pooled adjusted HR of 1.43 (95% CI: 1.25–1.64; *P* < 0.001). Because all included studies were observational, this finding represents an epidemiological time-to-event association and does not demonstrate that HF directly causes suicide death.

The evidence concerning suicide attempts was substantially more limited. Only two studies evaluated this outcome, and they differed markedly in population, HF severity, clinical setting, and effect measure. They were therefore summarized separately and were not combined with the suicide-mortality studies or with each other. Accordingly, the pooled HR from the primary analysis applies specifically to suicide mortality and should not be generalized to suicide attempts or suicidal ideation.

Between-study heterogeneity in the suicide-mortality meta-analysis was substantial (I^2^ = 83.0%). Potential sources include differences in healthcare systems, data sources, age and sex distributions, baseline psychiatric history, HF severity, ejection fraction phenotype, NYHA functional class, comorbidity and frailty burden, follow-up duration, mortality ascertainment, and covariate adjustment. The geographic and methodological distribution of the evidence was also highly imbalanced. Because only one study was conducted in Asia and only one study used a prospective design, subgroup comparisons were not performed. Furthermore, the small number of studies precluded reliable statistical assessment of publication bias or small-study effects.

### 4.2 Interpretation of findings and potential explanatory pathways

The observed association between HF and suicide mortality is likely multifactorial, while the evidence concerning suicide attempts remains insufficient for a common quantitative interpretation. However, the present meta-analysis cannot determine whether HF itself directly increases suicide mortality, whether suicide-related vulnerability arises from the consequences of advanced cardiovascular disease, or whether the association is explained by shared risk factors such as depression, anxiety, previous self-harm, psychiatric illness, alcohol or substance use, socioeconomic adversity, multimorbidity, frailty, cognitive impairment, chronic pain, social isolation, limited healthcare access, or palliative care needs. Therefore, the following biological and psychosocial explanations should be interpreted as hypothesis-generating rather than as mechanisms demonstrated by the included studies.

Several biological pathways may plausibly contribute to the observed association (19). HF is often accompanied by systemic inflammation, neurohormonal activation, autonomic dysfunction, sleep disturbance, impaired cerebral perfusion, and cognitive decline (20). In broader psychiatric and cardiovascular literature, inflammatory markers such as IL-6, TNF-α, and CRP have been linked to depressive symptoms, neuroinflammation, altered tryptophan metabolism, and suicidal behaviour (6). Cerebral hypoperfusion and cognitive impairment may also affect executive function, emotional regulation, and decision-making, which are relevant to suicidal vulnerability (21). Nevertheless, the cohort studies included in this meta-analysis did not directly measure inflammatory biomarkers, neurochemical alterations, cerebral perfusion, autonomic function, or cognitive trajectories, nor did they conduct mediation analyses. These pathways therefore remain biologically plausible but unconfirmed explanations for the epidemiological association observed in this study.

Psychosocial factors may also contribute to suicide-related vulnerability among patients with HF (22). Symptoms such as dyspnea, fatigue, exercise intolerance, orthopnea, and sleep disruption may reduce quality of life and functional independence (23). Recurrent hospitalizations, uncertainty about prognosis, financial strain, caregiver dependence, and perceived burdensomeness may further intensify psychological distress (24). Constructs from the Interpersonal Theory of Suicide, including perceived burdensomeness and thwarted belongingness, may be relevant in some patients with advanced HF (7). However, these factors may act as mediators, confounders, or consequences of disease progression, and the aggregated data available in this meta-analysis do not allow these roles to be disentangled.

Beyond depression, systemic inflammation, and psychosocial burden, HF should also be considered within a broader cardiovascular vulnerability framework. HF commonly coexists with multimorbidity, frailty, arrhythmic burden, autonomic imbalance, cognitive impairment, polypharmacy, recurrent hospitalization, and repeated exposure to acute cardiovascular events (25). Psychiatric manifestations in advanced cardiovascular disease may therefore reflect the cumulative interaction of these biological, clinical, and psychosocial vulnerabilities rather than depressive symptoms or inflammatory pathways alone. Emerging evidence from other cardiovascular populations supports this broader perspective. Studies of stress-related cardiomyopathies, such as Takotsubo syndrome, have highlighted bidirectional links between severe psychological distress, acute cardiovascular dysfunction, and adverse psychiatric outcomes, whereas studies in atrial fibrillation have demonstrated complex interactions among arrhythmia-related symptoms, cardiovascular disease burden, mental health, cognitive dysfunction, and long-term outcomes (26, 27).

In the context of HF, suicide-related vulnerability may be most pronounced among patients with advanced disease, recurrent decompensation, multimorbidity, frailty, cognitive impairment, atrial or ventricular arrhythmias, device therapy or LVAD support, poor social support, prior psychiatric history, or high treatment burden (28). These factors may confound, mediate, or modify the association between HF and suicide-related outcomes, but the aggregated data available in this meta-analysis do not allow these pathways to be disentangled. Future prospective studies should incorporate detailed cardiovascular phenotyping, psychiatric assessment, cognitive evaluation, and social vulnerability measures to identify high-risk HF phenotypes and clarify the mechanisms underlying the observed association (29).

From a clinical perspective, the observed association with suicide mortality supports greater awareness of psychological distress and suicide vulnerability in HF care (30). However, the present findings do not establish that routine screening or specific psychiatric interventions reduce suicide mortality or suicide attempts. Assessment of depression, psychological distress, and suicidal ideation may be considered as part of comprehensive HF management, particularly among patients with advanced disease, recent hospitalization, cognitive impairment, limited social support, or prior psychiatric history. Prospective and interventional studies are required to determine whether systematic screening, collaborative cardio-psychiatric care, or targeted psychosocial interventions improve mental health or suicide-related outcomes.

### 4.3 Strengths and limitations

This study has several strengths. First, it systematically evaluated longitudinal cohort evidence from more than 17 million participants. Second, only adjusted estimates were included, reducing, although not eliminating, the influence of measured confounding. Third, the revised outcome-specific analytical framework avoided statistically combining suicide mortality with suicide attempts and restricted the primary meta-analysis to studies reporting adjusted HRs for fatal outcomes. Fourth, study quality was systematically assessed using the NOS, and leave-one-out sensitivity analysis was conducted for the primary suicide-mortality meta-analysis.

However, the findings of this study must be interpreted in light of several important limitations. First, all included studies were observational, and therefore this meta-analysis can establish only an association rather than a causal relationship. Although most included cohorts adjusted for important covariates such as age, sex, psychiatric history, socioeconomic factors, and comorbidities, the degree and composition of adjustment varied substantially across studies. Residual or unmeasured confounding remains a major concern. Factors such as HF severity, NYHA functional class, ejection fraction phenotype, frailty, cognitive impairment, pain, alcohol or substance use, social isolation, financial toxicity, treatment burden, healthcare access, and palliative care involvement were not uniformly measured or adjusted for. These factors may partly explain the observed association between HF and suicide mortality and may also influence the individual estimates reported for suicide attempts.

Second, as a meta-analysis of aggregated literature-level data, we lacked access to individual patient data (IPD), which precluded us from performing more granular subgroup analyses based on disease severity and specific clinical phenotypes. Heart failure is a highly heterogeneous syndrome. It is biologically and psychologically plausible that the risk of suicide differs significantly between patients with preserved (HFpEF) vs. reduced ejection fraction (HFrEF), or among different NYHA functional classes. Specifically, end-stage HF patients (NYHA class III-IV) experiencing refractory symptoms, severe physical frailty, and chronic pain may face a disproportionately higher risk of suicide compared to those with stable, well-compensated disease. The inability to stratify risk by these precise clinical parameters limits our understanding of the exact dose-response relationship between illness severity and psychological burden. Therefore, potential pathways involving systemic inflammation, neurochemical alterations, autonomic dysfunction, cerebral hypoperfusion, sleep disturbance, perceived burdensomeness, or functional decline should be interpreted as plausible hypotheses rather than mechanisms demonstrated by this meta-analysis. Individual patient data, repeated psychiatric assessments, biomarker measurements, cognitive testing, and formal mediation analyses are needed to clarify whether these factors mediate, confound, or modify the association between HF and suicide-related outcomes.

Third, between-study heterogeneity remained substantial in the five-study suicide-mortality meta-analysis (I^2^ = 83.0%). This heterogeneity may reflect differences in healthcare systems, data sources, patient populations, HF severity, follow-up duration, mortality ascertainment, and covariate adjustment. Formal meta-regression was not performed because only five studies were available and such analyses would be underpowered and unstable. Although leave-one-out analysis assessed the influence of individual studies, it could not explain the underlying clinical and methodological heterogeneity. The pooled HR should therefore be interpreted as an average time-to-event association across heterogeneous observational cohorts rather than as a uniform estimate applicable to all HF populations.

Fourth, the available evidence covered clinically distinct outcomes. We addressed this limitation by separating suicide mortality from suicide attempts and by not calculating an overall estimate across these categories. Nevertheless, differences remained in how suicide death or completed suicide was defined and ascertained across mortality registries. Evidence regarding suicide attempts was limited to two clinically dissimilar studies, and no included study provided suicidal ideation as an independently extractable longitudinal outcome. Therefore, the results of the mortality meta-analysis cannot be generalized to nonfatal suicide attempts or suicidal ideation.

Fifth, the two studies evaluating suicide attempts reported different effect measures, namely an HR and an RR. Because these measures are not statistically interchangeable and the study populations were markedly different, we did not pool them. This decision avoids an inappropriate common estimate but also means that the magnitude and consistency of the association between HF and suicide attempts remain uncertain.

Sixth, the small number of studies prevented a reliable assessment of publication bias or small-study effects. We did not perform funnel plot asymmetry testing, Egger's regression, or trim-and-fill analysis because these methods may produce unstable or misleading findings when applied to only five heterogeneous studies. Publication bias and selective reporting therefore remain possible but could not be reliably quantified.

Finally, the geographic and methodological distribution of the evidence was highly imbalanced. Six studies were conducted in Europe and only one in Asia, while only one study used a prospective design. We therefore did not perform subgroup analyses by region or study design. The generalizability of the findings beyond predominantly European retrospective cohorts remains uncertain.

## 5 Conclusion

This systematic review and outcome-specific meta-analysis suggests that HF is associated with an increased hazard of suicide mortality based on five observational cohort studies reporting adjusted HRs. This finding should not be interpreted as evidence that HF directly causes suicide death because residual confounding and between-study heterogeneity remain important concerns.

Evidence regarding suicide attempts was limited to two clinically and methodologically dissimilar studies and did not support a reliable pooled estimate. No independently extractable longitudinal evidence was available for suicidal ideation. In addition, the small number of eligible studies precluded meaningful subgroup comparisons and reliable statistical assessment of publication bias. Further prospective studies using standardized outcome definitions, detailed HF phenotyping, comprehensive psychiatric and social assessments, and consistent effect measures are needed to clarify the associations of HF with suicide mortality and suicide attempts.

## Data Availability

The original contributions presented in the study are included in the article/Supplementary Material, further inquiries can be directed to the corresponding author/s.
