# Supplementary material for: Heart failure and suicide mortality and suicide attempts: a systematic review and meta-analysis of cohort studies
Source: Front Cardiovasc Med. 2026 Aug 28;13:1888860. doi: 10.3389/fcvm.2026.1888860 (PMC13561996; doi:10.3389/fcvm.2026.1888860)
Supplement: Supplementary file 1 [file Datasheet1.docx]

**Supporting information**

**Supplementary Figures**

**Supplementary Figure 1.** Leave-one-out sensitivity analysis of the association between heart failure and suicide mortality. Each circle represents the random-effects pooled hazard ratio after omission of the indicated study, and the horizontal line represents the corresponding 95% confidence interval. The vertical line indicates the overall pooled hazard ratio from the primary meta-analysis.

**Supplementary Tables**

**Table 1** The full search strategy of PubMed (2026.04.11)

| No. | Query | Results |
| --- | --- | --- |
| 1 | "Heart Failure"[Mesh] | 164,893 |
| 2 | ((Cardiac Failure[Title/Abstract]) OR (Heart Decompensation*[Title/Abstract])) OR (Congestive Heart Failure[Title/Abstract]) | 59,523 |
| 3 | ("Heart Failure"[Mesh]) OR (((Cardiac Failure[Title/Abstract]) OR (Heart Decompensation*[Title/Abstract])) OR (Congestive Heart Failure[Title/Abstract])) | 198,233 |
| 4 | "Suicide"[Mesh] | 83,434 |
| 5 | Suicide*[Title/Abstract] | 91,912 |
| 6 | ("Suicide"[Mesh]) OR (Suicide*[Title/Abstract]) | 118,529 |
| 7 | (("Heart Failure"[Mesh]) OR (((Cardiac Failure[Title/Abstract]) OR (Heart Decompensation*[Title/Abstract])) OR (Congestive Heart Failure[Title/Abstract]))) AND (("Suicide"[Mesh]) OR (Suicide*[Title/Abstract])) | 159 |

**Table 2** The full search strategy of Embase (2026.04.11)

| No. | Query | Results |
| --- | --- | --- |
| 1 | 'heart failure'/exp | 820,814 |
| 2 | 'cardiac failure':ab,ti OR 'heart decompensation*':ab,ti OR 'congestive heart failure':ab,ti | 87,513 |
| 3 | #1 OR #2 | 838,529 |
| 4 | 'suicide'/exp | 82,605 |
| 5 | 'suicide*':ab,ti | 115,058 |
| 6 | #4 OR #5 | 138,762 |
| 7 | #3 AND #6 | 1,658 |

**Table 3** The full search strategy of Cochrane library (2026.04.11)

| No. | Query | Results |
| --- | --- | --- |
| 1 | MeSH descriptor: [Heart Failure] explode all trees | 15,379 |
| 2 | (Cardiac Failure):ab,ti,kw OR (Heart Decompensation*):ab,ti,kw OR (Congestive Heart Failure):ab,ti,kw | 26,111 |
| 3 | #1 OR #2 | 34,329 |
| 4 | MeSH descriptor: [Suicide] explode all trees | 2,464 |
| 5 | (Suicide*):ti,ab,kw | 6,727 |
| 6 | #4 OR #5 | 7,013 |
| 7 | #3 AND #6 | 32 |
